# Unequal power relations and partner violence against women in Tanzania: a cross-sectional analysis

**DOI:** 10.1186/s12905-018-0675-0

**Published:** 2018-11-15

**Authors:** Seema Vyas, Henrica A. F. M. Jansen

**Affiliations:** 1 Department of Epidemiology and Biostatistics, Kilimanjaro Christian Medical University College, Moshi, PO Box 2240, United Republic of Tanzania; 2 Department of Population Health, London School of Hygiene and Tropical Medicine, Keppel Street, London, WC1E 7HT UK; 3 UNFPA Asia and the Pacific Regional Office (APRO), 4th Floor UN Service Building, Rajadamnoen Nok Avenue, Bangkok, 10200 Thailand

**Keywords:** Partner violence against women, Risk factors, Structural systems, Tanzania, Demographic and health surveys

## Abstract

**Background:**

Research on factors associated with partner violence against women is often framed within the context of gender inequality and power imbalances between husbands and wives—inequalities that are considered products of broader structural systems. Tanzania, a patriarchal society where high levels of partner violence exists, has gone through rapid economic and social changes over the past two decades. Increasing numbers of women are seeking paid work, and men’s ideals of manhood have reshaped with evidence of extra marital relations and alcohol use. Nationally representative population-based data documents 46.2% of ever-married women have experienced physical or sexual partner violence in their lifetime; 29.6% in the past year. In order to plan appropriate interventions to end violence against women, factors consistently associated with abuse need to be understood.

**Methods:**

This study uses “couples” data from the 2015 Tanzania Demographic and Health Survey to examine correlates of past year partner violence against women. Multivariate regression analysis was used to explore individual and relational-level variables—including socio-demographic characteristics and history of abuse among women, partner behavioural characteristics, and indicators of gender and economic inequality—among 1278 married and cohabiting couples.

**Results:**

At the individual level, women’s experiences of non-partner violence (sexual abuse by a non-partner and witnessing violence in childhood) was strongly associated with risk and highlights that all forms of violence against women serve to keep them subordinated. Partner behavioural characteristics (polygamy and problematic alcohol use) were also associated with risk. Household socio-economic status, however, was not significantly associated with women’s risk in the final multivariate model. At the relational-level, men’s age difference of 10 or more years; and any employment (compared to none/unpaid) were associated with lower risk. When considering attitudes tolerant towards wife abuse, the strongest association with risk of violence was when both partners held tolerant views.

**Conclusion:**

The findings support the assertions of violence being associated with women’s prior/additional experiences of abuse and with men’s harmful expressions of masculinity. In addition to interventions that focus on transforming gender norms and attitudes (at the individual and community levels), addressing economic, legal and political structural barriers are also required.

## Background

Violence against women is widely accepted as a human rights violation and public health concern [1, 2]. The most common form of violence against women is that perpetrated by men towards their female partners, and prevalence estimates suggest that globally, one in three women have experienced physical or sexual violence by an intimate partner (generally defined as a current or former spouse or cohabiting partner) in their lifetime [3].

Partner violence against women is more prevalent in patriarchal societies and research on associated risk factors is commonly framed within the context of unequal power relations that emphasise men’s and women’s roles, and assert men’s dominance over women [4, 5]. These gendered inequalities are theorised to be products of broader structural systems—political (e.g. lack of gender responsive policy making), legal (e.g. inadequate provision of legal and social services), and economic (e.g. unequal access to education, economic resources and employment opportunities)—that reinforce the disadvantaged status of women at both the community and the individual levels [6, 7].

Since the World Health Organization’s (WHO) 2000–2003 multi-country study on domestic violence and women’s health (WHO study) [1, 8], there has been an expansion of studies that have explored, besides prevalence and patterns of violence, male and female factors and their associations with partner violence against women. These studies have advanced understanding about the role of, in particular, individual level factors on gender relations and the mechanisms through which these factors shape women’s risk of partner violence.

Poverty or low household socio-economic status (SES) has been consistently found to be associated with higher rates of partner violence against women in low- and middle-income countries (LMIC) and in high income settings [9–12]. In a systematic review of published evidence from LMIC, in fifteen out of sixteen settings, household wealth (measured by ownership of durable assets) was found to have a protective association with women’s risk of past year physical or sexual violence, although, this association was significant in only eight settings and highlights that household wealth is not universally protective [9]. Early theories, *family stress theory,* argued that the inherent stress of poverty is the mediating factor that leads men to be violent towards their wives and female partners [11]. Poverty stress is further intensified in settings where ideals of successful manhood firmly place men to be the household’s main provider [12]. In such settings, limited or poor employment options for men may then lead to feelings of anxiety and despair and a crisis of male identity ensues. In some LMIC, including in Tanzania, it has been observed that feelings of economic disempowerment among men has resulted in a reshaping of masculine ideals that involve the excessive use of alcohol and relationships with other women, both of which have been found to significantly increase women’s risk of violence [5, 13–17].

The concept of successful manhood brings to the fore the tandem notion of “successful womanhood” that traditionally lay in reproductive responsibilities such as bearing children and especially sons, as well as in maintaining family values and family harmony [18, 19]. Transgressions of good or appropriate wifely behaviour include women’s use of alcohol, relationships with other men and displays of autonomy. While aspects of women’s empowerment such as education, economic independence and ownership of capital assets have been found to be protective in some settings, it has been found to have a risk association in others [9, 20]. Within the context of poverty, women’s financial contributions can ease financial stresses within households. A competing view, *relative resource theory,* however, asserts that economic (e.g. employment or income) or status (e.g. educational attainment) differentials that favour women over men increases a woman’s risk of violence because of challenges to established gender norms [21, 22]. So if a woman is working when her husband or male partner is not, then this may confer a risk onto women if this unequal status fuels men’s feelings of inadequacy [5, 21].

Another factor that has the effect to disadvantage women is early or other forms of abuse. Women’s early experience of violence (either childhood violence or witnessing their mother being beaten) may reinforce notions of inferiority and acceptance of abuse by a partner [5]. By contrast, men who witness violence towards their mothers or who were beaten themselves as children are more likely to become perpetrators of violence [17, 23, 24].

### Tanzania context

Tanzania has experienced steady economic growth as indicated by its Gross Domestic Product (GDP) which measured 7% (in 2016), a figure that has remained stable in the last decade [25]. By development indicators, the country made notable progress towards achieving the millennium development goal related to gender equality. By 2016 37% of national parliamentary seats were held by women [25], and in 2012 the ratio of girls to boys enrolled in primary school was almost parity (0.984), however, secondary schooling enrolment rate lagged behind (0.514) [26].

Despite this progress, Tanzania’s GDP per capita of $879US (in 2016) classifies the country as low income and the last poverty headcount ratio revealed that over one-quarter (28.2%) of its population live below the national poverty line (in 2011) [25]. Further, it remains a patriarchal society and high gender inequality continues to exist—with a gender inequality index score of 0.539 (in 2017), the country ranks 130 out of 159 [27]. Over two-thirds (68%) of Tanzania’s population reside in rural areas with small-scale agriculture the predominant livelihood for both men and women [25]. In addition to domestic duties, a very high proportion of women are engaged in productive work outside of the household, principally subsistence agricultural work in small farms (shamba) [25, 28].

During the late 1980s and early 1990s Tanzania embarked on a series of structural economic reforms, the effects of which led to rapid social changes [29]. Increasing numbers of men migrated from their natal home in search of employment opportunities that led to women taking on new roles such as responsibilities as the head of the household and seeking paid work [13, 30–32]. Social norms, however, continue to govern that men are the head of the household and the main family breadwinner, and women, whose responsibilities are familial, rely on their husbands for household needs [33–35]. As men faced increasing work insecurity and uncertain incomes, evidence began to emerge (in Dar es Salaam (DSM) and rural Kilosa, Morogoro Region) of men’s hostility towards women’s engagement in income earning activities [13, 35]. As women began to take on greater financial responsibilities, such as feeding the family, men began to retreat from theirs [13, 32]. In DSM high rates of abandoned women; extra-marital relationships; excessive drinking (among men) and frequent occurrence of aggression and violence between men and women were observed [13].

Prevalence estimates from the most recent (2015) Tanzania Demographic and Health Survey (DHS) confirms that partner violence against women is high—46.2% of ever-married women have experienced physical or sexual violence from their current or most recent partner in their lifetime; 29.6% in the past 12 months [36]—and is comparable to estimates from the 2010 Tanzania DHS when 43.6% of ever-married women (ages 15–49) reported they had experienced physical or sexual partner violence; 36.8% in the past 12 months [37].

While it is widely acknowledged that studies need to explore factors relating to both the woman and the man and the dynamics between them, often men’s characteristics are provided from the perspective of the woman. Using “matched couples” data from the 2015 Tanzania DHS, the objective of this study is to explore what factors are associated with women’s risk of past year partner violence.

## Methods

This study used the 2015 DHS data for “matched” couples in Tanzania. The DHS uses a multistage sampling method to select a nationally representative sample of households [36]. In the first stage 608 enumeration areas or “clusters” were selected with a probability proportional to size, from all 30 regions of the country (25 from mainland Tanzania and 5 from Zanzibar). Within each cluster, 22 households were randomly selected. A household survey, which included a listing of the names, ages and sex of all resident individuals, was administered and completed in 12,563 (of 13,360) households. In each selected household, a Woman’s questionnaire was administered to all eligible women (ages 15–49 and resident) from which one randomly selected woman received the module on domestic violence. In one-third of the selected households, all eligible men (ages 15–49 and resident) were interviewed using the Man’s questionnaire. In both questionnaires (Woman’s and Man’s), respondents were asked if they are married or living with someone as if married and if yes, the name of their partner. The respondent’s spouse/partner was then identified from the household listing and their unique household line number recorded. The DHS “couples” dataset links the two data files (women’s and men’s surveys) based on whom the respondents name as their partner.

**Fig. 1:**
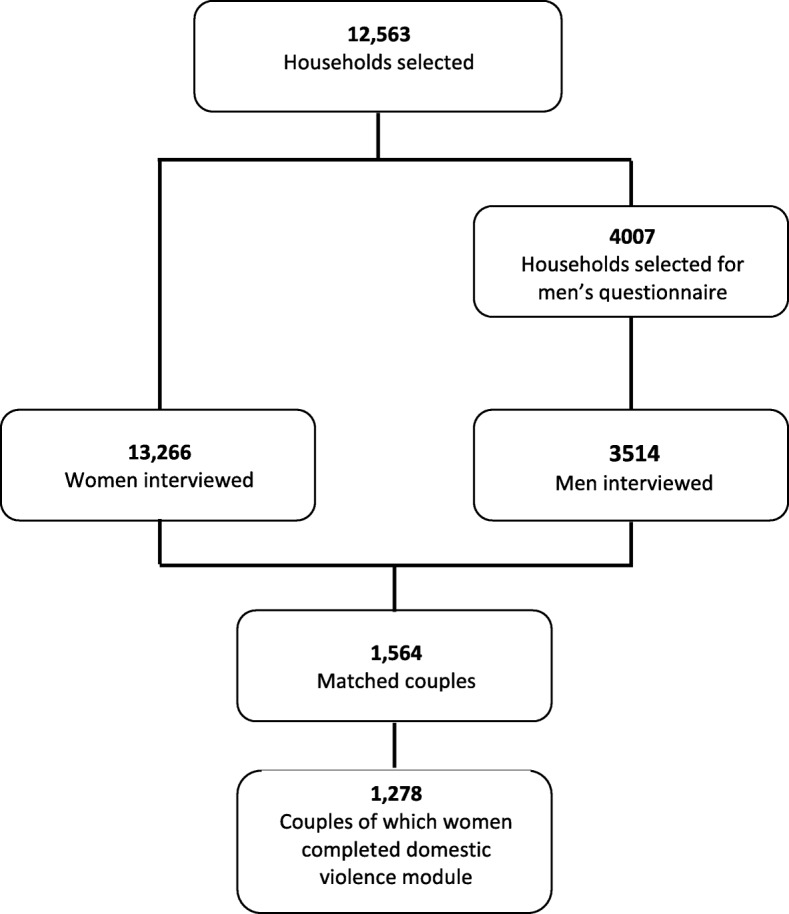
Sample of couples where both members completed the individual questionnaires (including the module on domestic violence)

Data were used for the 1278 matched couples where the domestic violence module had been administered to women (see Fig. 1). Verbal informed consent was obtained from all individual respondents included in the study who were able to accept or decline to participate.

### Partner violence against women

To measure physical or sexual partner violence, each woman was asked if her husband or partner had ever: pushed, shaken or thrown something at her; slapped her; twisted her arm or pulled her hair; punched her with his fist or with something that could hurt her; kicked, dragged or beat her; choked or burned her; threatened or attacked her with a knife or other weapon; physically forced her to have sexual intercourse when she did not want; physically forced her to perform other sexual acts; or forced her with threats to perform sexual acts. If a woman responded yes to any act, she was asked if it had happened in the past 12 months and a woman who gave an affirmative response to one or more act was considered to have experienced physical or sexual partner violence in the past 12 months.

### Covariates

Drawing on the conceptual framework on pathways to intimate partner violence developed by Jewkes [5] and on Heise’s ecological framework [38]—a theoretical and empirical-based schema that identifies known risk factors for partner violence against women—this study explores a total of twenty-five individual (women’s and men’s), household and relational variables in this analysis.

Among women’s socio-demographic characteristics, marital status and educational attainment were based on the DHS coding—educational attainment was based on the respondent’s years of schooling and coded into one of five categories (no education; incomplete primary; complete primary; incomplete secondary and complete secondary or higher). The variable *worked in the past year* is a composite variable based on women’s responses to three questions: whether the woman worked in the past 12 months; if yes, whether the work was either unpaid, paid in-kind or paid in cash; and whether the work was either seasonal or occasional or all year round (stable). Responses were combined to elicit the following four categories: not working/un-paid or paid in-kind (irrespective of whether the work was seasonal or stable)/seasonal paid in cash/stable paid in cash. Women’s ownership of capital assets was based on responses to two questions on ownership of a house or of land—both questions were recorded don’t own, owns alone or owns with someone in the DHS. Women who reported they owned at least one asset alone was coded as sole ownership, and women who reported joint ownership of one or both assets (but none alone) were coded joint ownership.

Women’s ages at first cohabitation and at first sex were recorded as a continuous variable in the DHS and subsequently categorised into age-groups for analysis. The number of children born to women, also recorded as a continuous variable, was capped at five.

Women’s attitudes towards wife-beating was based on the respondent’s acceptance of wife beating under at least one out of five circumstances—she goes out without telling him, she neglects the children, she argues with him, she refuses to have sex with him, and she burns the food—from which a binary no reason to hit/at least one reason to hit variable was created. A binary variable coded none or infrequent alcohol use/frequent alcohol use was based on responses to alcohol use in the past 12 months. Women reporting no use or less than once a month were considered infrequent users and women who reported they drank alcohol either every day or some days per month were considered frequent users.

Three binary no/yes variables to reflect women’s experiences or exposure to non-partner violence were created from responses to experiencing physical violence by a non-partner since the age of fifteen; to experiencing sexual violence by a non-partner either in childhood or as an adult; and to whether the respondents mother had been hit by her father.

Household socio-economic status (SES) was recorded as a five-category variable in the DHS and was based on household responses to ownership of assets and housing characteristics.

Among men’s characteristics, educational attainment, employment status, attitudes towards wife-beating, and frequent alcohol use were conceptualised in the same way as for women—although for men’s employment status the category not working was combined with un-paid or paid in-kind because of low counts (*n* = 6 men were not working). Men’s ages were categorised into three 10-year groups. Men were asked the number of wives/partner they had and the number of women they had fathered children with. For each question a binary variable was created to establish whether the man was in a polygamous relationship and whether he had fathered children with more than one woman.

Five relational-level variables, all measured on a four-level categorical scale, which reflect the extent of differences in the characteristic between the man and the woman, were also considered for analyses. Relative age was recorded as both having the same age if the age difference was less than five years/the man is older than the woman by 5–9 years/the man is older than the woman by 10 or more years/and the woman is older than the man by five or more years. Based on men’s and women’s recorded educational attainment, relative education was measured both have no education/both have the same level of education/the man achieved higher educational attainment/and the woman achieved higher educational attainment. Relative employment was conceptualised as both not working or are unpaid/both have the same type of employment (i.e. both are in seasonal paid employment or both are in stable paid employment)/the man has a higher level of employment (i.e. the man is in paid work and the woman is not working or the man is in stable paid work and the woman is either in seasonal paid work or not working)/the woman has a higher level of employment. Relative attitudes was coded as both partner’s express non-accepting attitudes towards wife-beating; both partner’s agree that wife-beating is acceptable under at least one out of five circumstances; the man agrees that wife-beating acceptable but the woman does not; the woman agrees that wife-beating is acceptable but the man does not. Finally, relative alcohol use was based on men’s and women’s self-reported frequency of alcohol use and coded both the man and woman did not drink alcohol or were infrequent users of alcohol/man only a frequent user/woman only a frequent user/and both frequent users.

### Data analysis

All analyses were conducted using STATA version 13.0 and adjusted for clustering. For the univariate logistic regression a *p*-value of less than 0.1 was considered significant and the variable retained for inclusion in the intermediate multivariate logistic regression (not shown) from which “significant” factors, i.e. had a p-value of < 0.1, were included in a final model. Two sets of multivariate regressions were run, the first only included individual level factors and the second included (in addition to the individual factors) the relational-level variables (where associated individual-level variables were removed because of collinearity). All regression results were adjusted for women’s age (continuous variable) and urban/rural location. In addition, for all regression analyses, respondents who had experienced physical or sexual partner violence in their lifetime but not in the past 12 months (*n* = 93) were excluded so as not to dilute associations [17].

## Results

### Respondent socio-demographic characteristics

The total number of couples in this study is 1278 (or 2556 individuals). Almost three-quarters of couples (73.2%) were married and 26.8% were living together as though married, however, 11% of men had more than one wife. Women’s mean age was 29.3 years (Std. Dev. 7.3) and men’s mean age was 34.9 years (Std. Dev 7.4). Over half (53.8%) of female respondents had completed primary schooling, however, fewer than one in five women (17.8%) had some secondary education or higher. Similar educational attainment levels were reported among men—54.4% had completed primary schooling and 18.5% had some secondary or higher education. The vast majority of women (82.9%) are in productive work—slightly over one in three women were unpaid or paid in kind; 22.5% were in seasonal paid work; and one-quarter were in stable paid work. Virtually all men were engaged in productive work—55.9% were in seasonal or unpaid work and 43.7% were in stable paid work.

Almost 40% of women (39.7%) reported they had experienced physical or sexual violence by their partner in their lifetime and one-third (33.1%) reported that they had experienced this in the past 12 months, thus highlighting that for few women, the violence had ceased.

### Prevalence of past year physical or sexual violence by covariate

Prevalence of past year partner violence was highest among women who were working either seasonally (39.8%) or who were in unpaid or paid in-kind work (36.2%); and who owned a capital asset either joint (39.1%) or alone (36.2%) (Table 1). Past year physical or sexual partner violence was also higher among women possessing attitudes tolerant towards wife abuse (agree with at least one reason to hit) compared to women who did not agree with any reason a man was justified to hit his wife (39.7% vs. 23.3%); who used alcohol frequently in the past 12 months compared to those who did not (54.4% vs. 31.4%) and among women with lower ages at first cohabitation (37.7%) and age at first sex (37.3%). Finally, prevalence of past year partner violence was higher among women with prior history of violence: non-partner physical violence (41.3%); non-partner sexual violence (49.2%); and mother hit by father (44.6%). Violence was lowest among women who had completed secondary education or higher (22.3%); who reported they had no births (26.2%); and who resided in the richest households (20.9%).

**Table 1:** Sample characteristics and OR (adjusted for woman’s age and urban/rural location) with past year physical or sexual violence

		*N*	% Past year Violence	OR	*p*-value
Woman’s characteristics					
Married	Married	936	31.7	1	
	Cohabiting (not married)	342	33.6	1.14	0.487
Education	No education	205	38.0	1	
	Incomplete primary	170	43.3	1.29	0.422
	complete primary	688	31.8	0.73	0.161
	Incomplete secondary	88	27.7	0.52	0.202
	Complete secondary or higher	127	22.3	0.51	0.055
Working	Not working	219	28.0	1	
	Unpaid/ in-kind	453	36.2	1.37	0.233
	Seasonal paid	287	39.8	1.68	0.056
	Stable paid	319	26.2	0.96	0.890
Capital assets	Doesn’t own	551	26.3	1	
	Owns alone	307	36.2	1.85	0.003
	Joint ownership	420	39.1	2.04	< 0.001
Parity	None	112	26.2	1	
	1	213	30.9	1.51	0.240
	2	273	33.2	1.94	0.051
	3	234	35.4	2.51	0.012
	4	159	35.2	3.21	0.007
	5 or more	287	34.6	3.67	0.007
Age at first cohabitation	21 years or more	310	27.2	1	
	17 years or less	508	37.7	1.57	0.037
	18–20 years	460	31.1	1.16	0.463
Age at first intercourse	18 years or more	442	23.9	1	
	17 years or less	836	37.3	1.85	0.001
Attitudes	No reason to hit	535	23.3	1	
	At least one reason to hit	743	39.7	2.08	< 0.001
Alcohol use	None or infrequent use	1185	31.4	1	
	Frequent use	93	54.4	3.16	< 0.001
Non partner physical violence	No	1193	32.2	1	
	Yes	79	43.4	1.58	0.173
Non partner sexual violence	No	1217	32.2	1	
	Yes	61	49.2	2.82	0.001
Mother hit	No / DK	823	26.2	1	
	Yes	455	44.6	2.47	< 0.001
Household characteristics					
Household SES	Poorest	246	37.9	1	
	Poorer	238	35.1	0.93	0.782
	Middle	268	35.3	0.95	0.866
	Richer	296	34.8	0.95	0.856
	Richest	230	20.9	0.50	0.034
Man’s characteristics					
Age	20–29	339	37.1	1	
	30–39	575	33.7	0.95	0.830
	40–49	364	28.7	0.79	0.511
Education	No education	140	34.6	1	
	Incomplete primary	206	36.4	1.16	0.643
	complete primary	695	33.1	1.02	0.950
	Incomplete secondary	91	33.3	0.97	0.935
	Complete secondary or higher	146	27.9	0.94	0.850
Working	Unpaid / In-kind^a^	173	53.0	1	
	Seasonal paid	485	33.2	0.42	< 0.001
	Stable paid	619	28.5	0.37	< 0.001
Attitudes	No reason to hit	868	28.6	1	
	At least one reason to hit	410	41.8	1.90	< 0.001
Polygamy	No	1138	31.2	1	
	Yes	140	47.0	2.03	0.008
Fathered children	None or one woman	841	30.8	1	
	More than one woman	436	37.6	1.39	0.049
Frequent alcohol use	No	968	27.3	1	
	Yes	287	50.3	1.96	0.001
Relational factors					
Relative age	Same	556	35.0	1	
	Him older 5–9 years	468	33.7	0.86	0.415
	Him older 10+ years	234	27.7	0.58	0.010
	Her older 5+ years	20	30.0	0.88	0.836
Relative education	Both no education	58	42.2	1	
	Both same	590	30.9	0.73	0.393
	His higher	347	37.0	1.03	0.945
	Hers higher	283	30.7	0.69	0.336
Relative employment status	Both unpaid/ not working	111	53.9	1	
	Both same type	344	27.4	0.37	0.001
	Him higher	677	32.5	0.45	0.003
	Hers higher	145	36.0	0.53	0.057
Relative attitudes	Both no reason	395	20.5	1	
	Both reason to hit	270	46.7	3.53	< 0.001
	Him reason	140	31.3	1.82	0.039
	Her reason	473	35.3	1.99	0.001
Relative alcohol use	Neither use	940	29.5	1	
	Both used	63	57.1	4.70	< 0.001
	Him only	224	38.6	1.72	0.012
	Her only	28	49.9	2.44	0.031

^a^Includes Not working

Past year physical or sexual violence against women was higher in relationships where men were in unpaid or paid in-kind work (53.0%); men were polygamous (47.0%); men had children with more than one woman (37.6%); when men held attitudes tolerant towards wife-beating (41.8%); and when men reported frequent alcohol use compared to men who reported no or infrequent alcohol use (54.4% vs. 31.4%).

When considering relational-level factors, past year physical or sexual partner violence was highest in relationships when both displayed the characteristic that was associated with highest risk of violence at individual level, e.g. when both the woman and the man had no education (42.2%); were in unpaid work or not working (53.9%); possessed attitudes tolerant towards wife-beating (46.7%); and used alcohol frequently in the past 12 months (57.1%).

### Multivariate logistic regression analyses

In the final individual model, four women’s characteristics were significantly associated with higher risk of past year partner violence. Compared to women having no births, women reporting at least one birth had higher odds of experiencing past year partner violence—with significant associations found with having had 2 births. Women’s tolerant attitudes towards wife-beating, witnessing mother hit by father and early age at first sex were also significantly associated with higher odds of violence. Among the man’s characteristics, polygamy, attitudes tolerant towards wife-beating and frequent alcohol use were significantly associated with past year physical or sexual violence against women. Seasonal paid and stable paid work were both, however, associated with women’s lower risk of experiencing violence in the past year.

The result of the final relational model is also shown in Table 2. All four relational factors included in the multivariate model had responses that were significantly associated with women’s experiences of past year physical or sexual partner violence. An age difference where the man is 10 or more years older than the woman significantly reduced women’s odds of experiencing partner violence in the past year. Compared to households where both the man and woman were not working or in unpaid work, households where at least one of the partner was in paid work was associated with lower risk—significant lower risk being found when both the woman and man are in the same type (either both seasonal or both stable) of paid work, or when the man is in stable paid work and the woman in seasonal paid work.

**Table 2:** Final multivariate logistic regression models to identify factors associated with past year physical and sexual partner violence among currently married/cohabiting women

		Individual model			Relational model		
		AOR	95% CI		AOR	95% CI	
Woman’s characteristics							
Capital assets	Doesn’t own	1			1		
	Owns alone	1.47	0.95	2.30	1.38	0.91	2.11
	Joint ownership	1.51	0.98	2.33	1.50	0.99	2.28
Parity	None	1			1		
	1	1.55	0.78	3.08	1.49	0.76	2.93
	2	2.00	1.02	3.93	1.87	0.97	3.61
	3	1.89	0.93	3.87	1.45	0.76	2.77
	4	1.94	0.80	4.70	1.33	0.65	2.72
	5 or more	2.33	0.94	5.77	1.46	0.72	2.99
Age at first intercourse	18 years or more	1			1		
	17 years or less	1.51	1.00	2.26	1.72	1.14	2.58
Attitudes	No reason to hit	1					
	At least one reason to hit	1.65	1.14	2.39			
Non partner sexual violence	No	1			1		
	Yes	2.30	0.99	5.35	2.28	0.93	5.59
Mother hit	No/DK	1			1		
	Yes	2.22	1.58	3.13	2.25	1.61	3.13
Man’s characteristics							
Working	Unpaid/In-kind^a^	1					
	Seasonal paid	0.49	0.30	0.79			
	Stable paid	0.43	0.26	0.74			
Attitudes	No reason to hit	1					
	At least one reason to hit	1.57	1.12	2.19			
Polygamy	No	1			1		
	Yes	1.87	1.02	3.43	1.90	1.03	3.49
Frequent alcohol use	No	1					
	Yes	1.83	1.23	2.71			
Relational factors							
Relative age	Same				1		
	Him older 5–9 years				0.79	0.54	1.15
	Him older 10+ years				0.52	0.31	0.85
	Her older 5+ years				0.50	0.14	1.81
Relative employment status	Both unpaid/not working				1		
	Both same type				0.40	0.23	0.72
	Him higher				0.49	0.29	0.82
	Hers higher				0.59	0.30	1.14
Relative attitudes	Both no reason				1		
	Both reason to hit				2.84	1.75	4.60
	Him reason				1.94	1.03	3.64
	Her reason				1.83	1.18	2.84
Relative alcohol use	Neither use				1		
	Both used				3.25	1.44	7.36
	Him only				1.64	1.05	2.55
	Her only				1.33	0.58	3.03
Location	Rural				1		
	Urban				0.87	0.58	1.32

^a^Includes not working

Possessing attitudes tolerant towards wife abuse was significantly associated with higher risk of violence with the strongest association found in couples where both the woman and her partner held tolerant views. Likewise, with alcohol use, the strongest association with higher risk of partner violence was when both the woman and her partner reported frequent alcohol use.

## Discussion

Almost 40% of women in this “matched” sample reported that they had experienced physical or sexual violence by an intimate partner in their lifetime and past year prevalence was 33%. These prevalence estimates have not notably changed since the first population-based prevalence estimates documented by the WHO (in 2002) when 40% of currently partnered women in Dar es Salaam and 55.6% in Mbeya reported lifetime experience of physical or sexual partner violence and 20.8 and 31.0% reported partner violence in the past year [39].

Several key factors were found to influence women’s risk of experiencing violence at the hands of their partners. Among factors relating to the woman, a significant risk association was found with exposure to violence in childhood (witnessing mother being hit). Intergenerational exposure to violence has been consistently found to increase women’s risk of partner violence in studies from Tanzania and in studies across cultures [16, 40]. This highlights the cyclical nature of violence that serve to disadvantage women by conferring vulnerability in later life. Tolerant attitudes towards wife beating, an indicator of women’s low social value and the extent of male hierarchy that exists within society, measured from the perspectives of the woman and the man, were found to increase women’s odds of experiencing partner violence, and in the relational model, the strongest association with violence was found when both partners held tolerant views. Women’s young age at sex initiation (17 years or lower) was also found to elevate women’s risk of past year partner violence. A study in Rakai District, Uganda, documented the same result and argued either early sexual onset has a disempowering effect on women who are then less able to protect themselves against violence later in life, or that women who become sexually active early are self-selected for subsequent abusive relationships [41].

Having given birth to one or more children elevated women’s risk of past year partner violence compared to not having given birth, although interestingly, the strong increase with higher parity that was seen in the bivariate analysis does not remain in the individual or relational models. A study in Viet Nam documented similar findings in terms of having children or not [17]. It could be hypothesized that there is a potential link between increasing number of children and a decrease in potential for women to engage in employment, and that the observed association is a reinforcement of the structural norms which serve to keep women dependent on their partners. Some studies, however, have suggested that high parity and unintended pregnancy is a consequence of violence, rather than a risk factor for violence, and is related to women’s lack of ability to control their fertility [42, 43].

Although marginally insignificant in the multivariate model, the risk association with women’s sole ownership of land and/or housing, which was significant at the bivariate level, is counter to the supposition from economic theory that ownership of capital assets empowers women to negotiate less violence—as has been found in Kerala, India [44]. Women’s independent wealth is hypothesised to raise women’s bargaining power or to facilitate women’s ability to leave a violent relationship by lowering their “threat point”—the threshold at which a woman evaluates that her welfare is better outside of the household [45]. Analysis of the WHO study data from Tanzania found that ownership of capital assets did not have the empowering effect to enable abused women to leave a violent relationship [46]. Exactly why ownership of a capital asset increases women’s risk of partner violence is not clear. It may be that women who own assets are more likely to be confrontational or that it is a transgression of gender norms and men use violence to reassert their dominance within the household. Alternatively, it may be that abused women who are able to, invest in capital assets with the view to leave a violent relationship at some point in the future. Greater household SES, usually measured by ownership of assets in LMIC, has generally been found to be protective (although not always significantly) [9, 16]. In this study, a significant protective association with asset wealth was found only at the bivariate level and with only the highest asset quintile. This could reflect a greater difference in wealth between the top one-fifth of households and the rest.

Among men’s characteristics, polygamy and alcohol use—expressions of masculinity that have increased with the structural economic reforms and work insecurity—were both significantly and positively correlated with women’s risk of partner violence, and both factors have been consistently found to be associated with women’s risk of violence in Tanzania and elsewhere [14, 17, 39, 47]. Reasons for the finding with polygamy could be that women have less power and are more likely to be dependent on their husbands, thus raising their vulnerability, or because women are likely to chafe at this expression of traditional privileges, as found in Moshi, Tanzania [50]. While several theories have been advanced to explain the relationship between men’s alcohol use and partner violence against women, an in-depth analysis from fourteen sub-Saharan Africa countries concluded that the most likely causes for the significant correlation (in all fourteen countries) were behavioural disinhibition—that alcohol use impairs cognitive functioning and increases aggression—and relationship dissatisfaction [48]. Although an independent risk factor by itself, men’s excessive alcohol use in Tanzania has been argued to have arisen in part because of men’s increasing frustrations at not being able to meet their gendered role expectations as the household’s main breadwinner [12].

In this study, compared to not working, the odds of partner violence against women was significantly lower when the man was working and a lower odds ratio (albeit slightly) was observed among men in stable paid work. Interestingly, and in line with family stress theory, in the relational model both the woman and the man working in the same category of employment (either both stable paid or both seasonal paid) and when the man is in stable paid and the woman in seasonal employment (i.e. the man has higher employment status), reduced the odds of women experiencing violence.

Several limitations underlie this study which must be borne in mind when interpreting the results. The first is that the cross-sectional design of the study means that it is not possible to determine the directionality of relationships between many of the variables and partner violence. A second limitation of this study is that the analysis is limited by the variables provided which have not necessarily been collected with this analysis in mind. For example, some studies highlight an even higher risk associated with the male partner’s witnessing or experiencing violence as a child (compared to the woman) [17, 23, 24], but our dataset did not include variables that enabled looking at this. Further limitations to highlight include that the DHS data collection may not train researchers to collect sensitive data on partner violence in the same robust way as studies like the WHO study; or that couples who took part in the DHS surveys are different from those where one or both partner declined to take part; and finally, the analysis does not use “dyadic analytical techniques” to take into consideration that individuals are linked, but rather constructed simplistic couple-level indicators.

## Conclusion

Over the last few years, Tanzania society has undergone structural economic reforms, with an increased number of women working and an increased number of men experiencing a crisis of masculinities. At the same time, the government of Tanzania has introduced measures to address violence against women in the country with developments of guidelines for clinical management and law enforcement [49]. Against this backdrop, prevalence of partner violence against women remains high. In other LMIC, targeted interventions to individuals, couples and the wider community have been found to reduce rates of partner violence [50]. Greater efforts, however, are required to address the structural constraints that perpetuate gender inequalities and that will continue to put women at risk. As efforts to address violence against women go on in Tanzania, this study provides an invaluable benchmark for the continuous monitoring of the effects these scale-up attempts have on reducing incidence of violence against women.

## Abbreviations

DHS: Demographic and Health Survey
GDP: Gross Domestic Product
LMIC: low- and middle-income countries
SES: Socio-economic status
WHO: World Health Organization
